# Sealing ability of MTA, CPM, and MBPc as root-end filling materials: a bacterial leakage study

**DOI:** 10.1590/1678-775720130103

**Published:** 2016

**Authors:** Paulo Leal MEDEIROS, Norberti BERNARDINELI, Bruno Cavalini CAVENAGO, Sérgio Aparecido TORRES, Marco Antonio Hungaro DUARTE, Clovis Monteiro BRAMANTE, Marina Angélica MARCIANO

**Affiliations:** 1 - Universidade de São Paulo, Faculdade de Odontologia de Bauru, Departamento de Dentística, Materiais Odontológicos e Endodontia, Bauru, SP, Brasil; 2 - Universidade de São Paulo, Faculdade de Odontologia de Bauru, Departamento de Microbiologia e Imunologia, Bauru, SP, Brasil

**Keywords:** Endodontics, Dental pulp cavity, Epoxy resins

## Abstract

**Objectives:**

To evaluate the sealing ability of three root-end filling materials (white MTA, CPM, and MBPc) using an *Enterococcus faecalis* leakage model.

**Material and Methods:**

Seventy single-root extracted human teeth were instrumented and root-ends were resected to prepare 3 mm depth cavities. Root-end preparations were filled with white MTA, CPM, and MBPc cements. *Enterococcus faecalis* was coronally introduced and the apical portion was immersed in BHI culture medium with phenol red indicator. The bacterial leakage was monitored every 24 h for 4 weeks. The statistical analysis was performed using the Wilcoxon-Gehan test (p<0.05).

**Results:**

All cements showed bacterial leakage after 24 hours, except for the negative control group. The MBPc showed significantly less bacterial leakage compared with the MTA group (p<0.05). No significant differences were found between the CPM and the other groups.

**Conclusions:**

The epoxy resin-based cement MBPc had lower bacterial leakage compared with the calcium silicate-based cements MTA and CPM.

## INTRODUCTION

Persistent microorganisms are the main factors associated with chronic apical periodontitis and consequent Endodontic failures^10^. One of the indications for periradicular surgery is when the conventional treatment is not able to eliminate the microorganisms of the apical portion^7^. These procedures involve the use of filling materials to prevent bacterial leakage and the reinfection of the root canal system^27^. Therefore, it is crucial for the cement used to obturate root-end cavities to be able to provide a tridimensional filling.

Several materials were suggested for retrograde fillings, e.g., amalgam, EBA, IRM, gutta-percha, and MTA^2^ ^,^ ^32^. The Mineral Trioxide Aggregate (MTA) is currently used in retrograde fillings, showing satisfactory physicochemical^15^ ^,^ ^29^ and biological properties^28^ ^,^ ^30^. Other cements are studied as alternatives for the MTA. In 2004, a novel root-end filling material comparable with the MTA was developed. The CPM (EGEO SLR, MTM Argentina SA, Buenos Aires, Argentina) is composed of a powder mainly made of Portland cement and a liquid with distilled water in its composition. The CPM cement presents antimicrobial activity^23^, satisfactory biological response^18^ ^,^ ^20^, and adequate physicochemical properties^4^ ^,^ ^24^.

Epoxy-resin based cements have been widely studied for Endodontic procedures since 1957^8^ ^,^ ^19^. In 1984, Moraes and Berbert developed an epoxy resin-based cement containing calcium hydroxide to be used as a retrograde filling material^13^. The MBPc is consisted of hydrophobic base paste/catalyst paste cement^14^. Promissory results regarding its physical^34^, chemical^14^ ^,^ ^33^, and biological^3^ ^,^ ^9^ properties were found. Nonetheless, this cement requires more investigation to be considered clinically useful.

There are several methods to evaluate the sealing ability of filling materials such as fluid infiltration^34^, radioisotope^22^, dye penetration^6^ ^,^ ^14^, and bacterial leakage^21^. Radioisotope and dye penetration methods present the disadvantage of having the molecular size of radioisotopes tracers and dye particles smaller than bacteria^31^. On the other hand, bacterial leakage is considered more clinically relevant^31^. This method is widely used in Endodontic research to evaluate the sealing ability of root canal sealers^12^ ^,^ ^26^ and root-end filling materials^1^ ^,^ ^11^. A previous study has compared MTA, CPM, and MBPc cements using a dye penetration model^14^. Considering the limitations of this method, it is indispensable for us to investigate if there is bacterial infiltration in root-end fillings with these cements. Thus, the aim of the study was to evaluate the sealing ability of three root-end filling materials (white MTA, CPM, and MBPc) using an *Enterococcus faecalis* leakage model.

## MATERIAL AND METHODS

Seventy intact, caries-free, single-rooted permanent human teeth were selected. The ethics committee of the Bauru School of Dentistry approved the use of teeth for research purposes (CEP 049-2007). All teeth were autoclaved. The coronal portions were sectioned 1.0 mm below the dental-enamel junction using a 0.3 mm Isomet saw (Buehler, Lake Bluff, Illinois, USA). Roots were standardized at 15 mm and the working length (WL) was established 1.0 mm from the apical foramen. Root canals were prepared using the crown-down technique with K-files (Dentsply Maillefer, Ballaigues, Switzerland). Apical preparation was performed at working length up to a size 40 K-file. The shaping procedure was completed with 2 and 3 Gates-Glidden drills (Dentsply Maillefer, Ballaigues, Switzerland). After the use of each instrument, we irrigated the canal with 2 mL of 2.5% sodium hypochlorite. The smear layer was removed with 2 mL of 17% EDTA (Biodinâmica, Ibiporã, Paraná, Brazil) for three minutes, then a 2 mL flush of distilled water was used as the final rinse and the canals were dried with paper points (DentsplyMaillefer). The final 3.0 mm of each root was resected with a cylindrical diamond bur, perpendicularly to the long axis of the root. A root-end cavity with 3.0 mm of depth and 1.0 mm of diameter was prepared. A gutta-percha cone of size 40.02 was inserted into the root canal 3 mm from the final of the root to provide an intracanal matrix to pack the root-end filling material against.

### Root-end filling

The specimens were randomly divided in three groups (n=20):

Group 1: MTA (Angelus, Londrina, Paraná, Brazil);

Group 2: CPM (Egeo, Buenos Aires, Argentine);

Group 3: MBPc.

**Figure 1:**
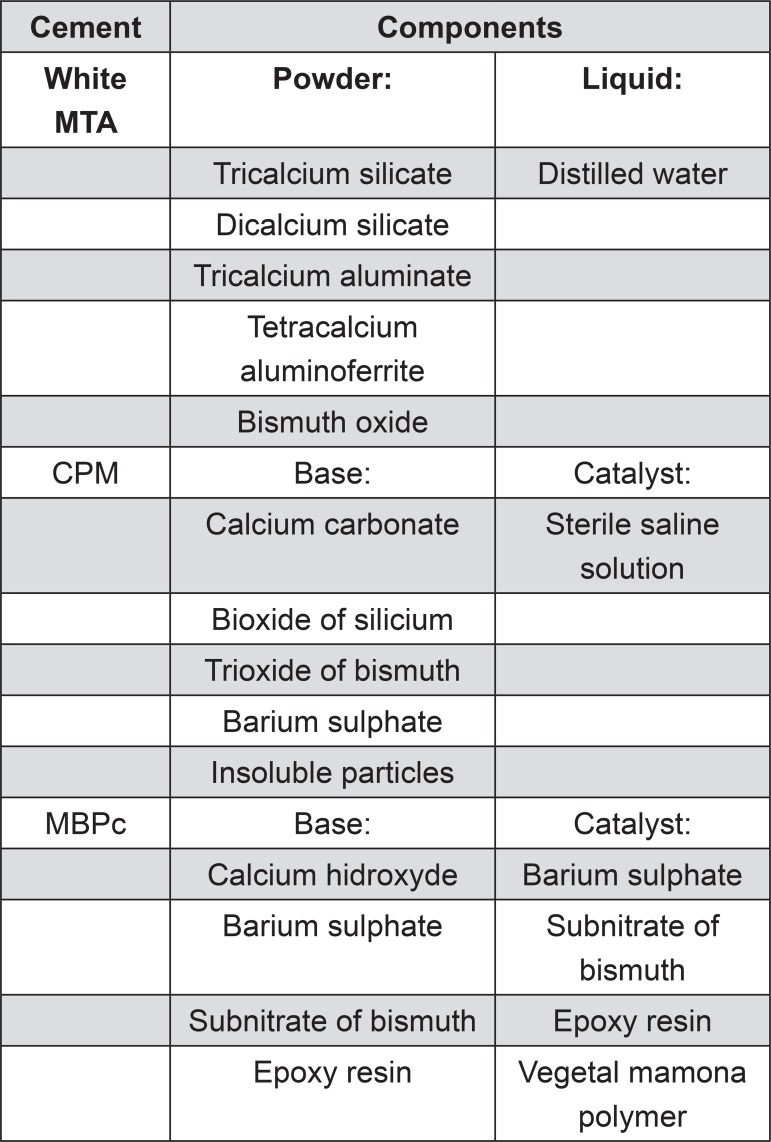
Composition of the evaluated cements

The composition of the evaluated cements is present in Figure 1. Cements were manipulated by following the manufacturer’s instructions. They were incrementally inserted into the root-end cavities and vertically compacted using a hand plugger. Specimens were stored in 100% of humidity at 37°C for one week to allow the cements to set. For negative control (n=5), the entire root surface was coated with two layers of nail varnish (Colorama, L’Oréal, São Paulo, São Paulo, Brazil) after filling all root-end cavities. Positive control (n=5) was left with unfilled root-end cavities.

### Bacterial leakage

**Figure 2:**
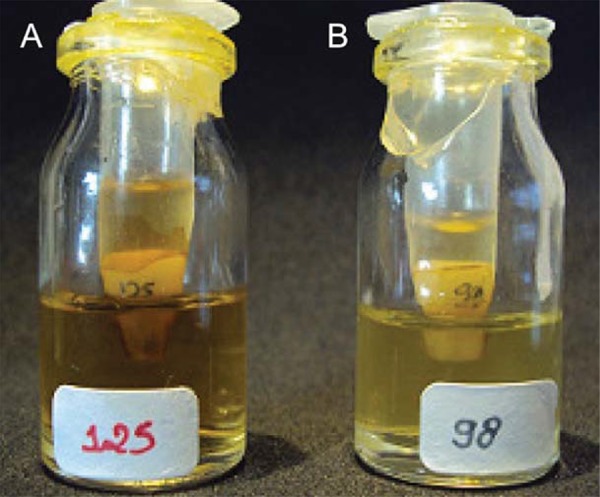
Representative leakage apparatus used for the experiment. Negative microbial growth is shown in A and positive microbial growth can be observed in B

The apparatus used to evaluate the leakage was prepared as previously described^21^. Glass vials with rubber stoppers were adjusted to use. Using a high-speed handpiece, a hole was made through the centre of each rubber stopper in which each tooth was inserted under a pressure up to 4 mm from the apical portion. An epoxy resin-based varnish (Araldite, Brascola, São Paulo, São Paulo, Brazil) was used to seal the interface between tooth and rubber stopper. Cylinders prepared with 10 mL plastic syringes were adapted to the outer surface of the stoppers to create a chamber around the crown of the tooth. The apparatus was sterilized in ethylene oxide gas for a 4 h cycle at 56°C (Figure 2).

The standard bacterial strains of *Enterococcus faecalis* (ATCC 29212) were used in the study. The Brain Heart Infusion Medium (BHI, Difco, BD Diagnostic Systems, Sparks, Maryland, USA) was used for bacterial growth. *Enterococcus faecalis* was inoculated in tubes containing 5 mL of sterile BHI suspension and incubated at 37^o^C for 24 hours. To adjust the experimental suspensions, turbidity of culture microorganisms were verified with an spectrophotometer (1105, Bel Photonics do Brasil Ltda., Osasco, Brazil) at 540 nm, bacterial cells were resuspended according to the MacFarland scale to obtain a final concentration of 3x10^8^ colony-forming units (CFU)/mL. The purity of the cultures was confirmed by the Gram staining method and the morphology of the colony.

Sterile pipettes were used to place 400 µl of bacterial inoculum in each access cavity found in the syringe apparatuses (in the upper region, removing the gauze stop). The 4 mm of root-ends were immersed in sterile BHI. The apparatus was incubated at 37°C for 120 days and checked daily for turbidity in the BHI broth. Bacterial leakage was considered when turbidity was observed (Table 1).

**Table 1:** Number of samples with positive bacterial leakage every 30 days

Cement	Total of samples	1-30 days	31-60 days	61-90 days	91-120 days	Total
White MTA	20	7	0	0	0	7
CPM	20	4	0	0	0	4
MBPc	20	1	0	1	0	2
Positive control	5	5	0	0	5	5
Negative control	5	0	0	0	0	0

### Statistical analysis

Statistical analysis was performed using the Wilcoxon-Gehan test to compare the evaluated groups (p<0.05).

## RESULTS

**Figure 3:**
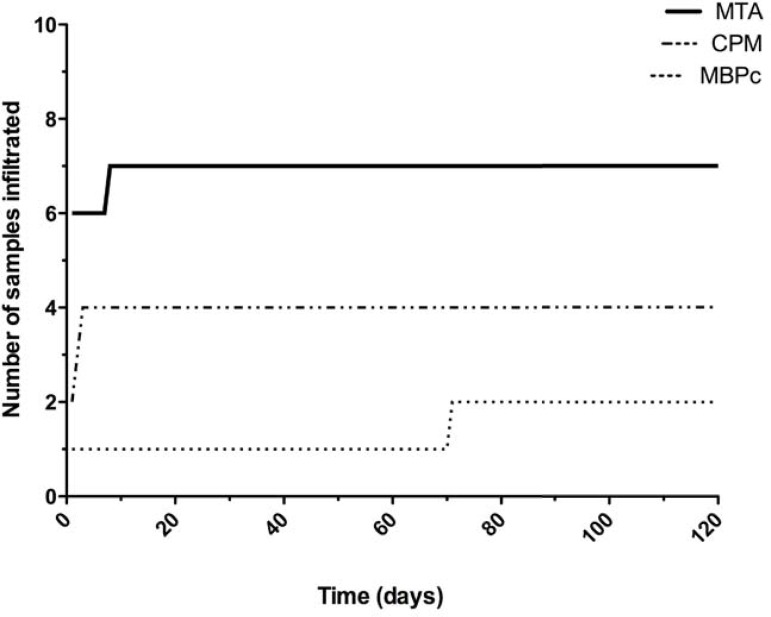
Number of samples leaked during a period of 120 days

The criteria evaluated with experimental periods are shown in Figure 3. All cements showed bacterial leakage after 24 hours, except for the negative control group. The MTA showed a higher number of specimens that leaked compared with the MBPc group (p<0.05). Statistical similarities for bacterial leakage were found between the CPM and the MBPc and also the MTA (p>0.05). After 31 days until the final period, only one specimen (MBPc) among all groups showed infiltration.

## DISCUSSION

The success of retrograde fillings concerns the properties of the cement used^17^. The aim of root-end filling materials is to adhere to the dentine of the apical portion difficulty bacterial leakage providing conditions to periapical healing^32^. The sealing ability of a cement can be determined using leakage methods^12^ ^,^ ^14^, confocal microscopy^32^, scanning electron microscopy^25^, and more recently micro-computed tomography analysis^35^. The bacterial leakage method has the advantage of providing a clinically relevant adaptability date of materials for root canal walls^31^.

The *Enterococcus faecalis* was chosen for this study model because it is commonly present in secondary infections from Endodontic treatment failures. This microorganism is a gram-positive coccus and is highly resistant to alkaline pH such as the one present in the calcium hydroxide paste. Because of its high incidence in filled root canals and their drug resistance, the *E. faecalis* has been proposed as an Endodontic pathogen^16^. Furthermore, this microorganism is easily arranged and interpreted from the study data^11^. Under the conditions of this study, this microorganism was able to leak in all experimental cements. On the other hand, Jacobovitz, et al.^5^ (2009), using another model of *in vitro* microleakage analysis with the same microorganism, found that the MTA showed no microbial growth after 30 days.

The results found in the present study showed that the bacterial leakage occurred after 24 hours in at least one specimen for MTA, CPM, and MBPc cements. During the experimental period, all groups showed decrease in the number of leaked specimens. A possible explanation is that the cements expanded into the cavities during the period and difficult the leakage. The results found in the present study are according to that previously found by Orosco, et al.^35^ (2008), although they have used the dye penetration method.

Although no statistical differences were found between MBPc and CPM cements (p>0.05), the MBPc showed statistically less infiltration compared with the MTA (p<0.05). In a previous study, the MBPc showed satisfactory results for marginal adaptation and leakage^14^. Vasconcelos, et al.^34^ (2011) reported low leakage ranges for epoxy-resin sealers MBPc and AH Plus compared with the MTA, using a fluid infiltration method. Possibly the presence of epoxy-resin improved the sealing of MBPc and AH Plus in dentin walls.

The modified Portland cement (CPM) contains calcium carbonate, silicon dioxide, bismuth trioxide, and barium sulphate^33^. Probably, the presence of calcium carbonate could be the responsible for offering a great calcium ions release, which may promote the adhesion to dentinal canal walls, thus improving sealing properties^33^. According to the results, this cement has not been able to inhibit bacterial leakage completely. The absence of studies evaluating the CPM makes difficult the comparison regarding its sealing ability.

Further studies should use the confocal laser scanning (CLSM) microscopy to detect and quantify bacterial viability in void spaces or gaps between cavity walls. Moreover, prospective clinical studies evaluating the success rate of Endodontic surgeries using the tested sealers might prove to be informative.

## CONCLUSION

The epoxy resin-based cement MBPc had lower bacterial leakage compared with the calcium silicate-based cements MTA and CPM.
